# Linkage disequilibrium in crossbred and pure line chickens

**DOI:** 10.1186/s12711-015-0098-4

**Published:** 2015-02-26

**Authors:** Weixuan Fu, Jack CM Dekkers, William R Lee, Behnam Abasht

**Affiliations:** Department of Animal and Food Sciences, University of Delaware, Newark, DE 19716 USA; Department of Animal Science, Iowa State University, Ames, IA 50011 USA; Heritage Breeders, LLC, Salisbury, MD 21804 USA

## Abstract

**Background:**

Both genome-wide association (GWA) studies and genomic selection depend on the level of non-random association of alleles at different loci, i.e. linkage disequilibrium (LD), across the genome. Therefore, characterizing LD is of fundamental importance to implement both approaches. In this study, using a 60K single nucleotide polymorphism (SNP) panel, we estimated LD and haplotype structure in crossbred broiler chickens and their component pure lines (one male and two female lines) and calculated the consistency of LD between these populations.

**Results:**

The average level of LD (measured by *r*^2^) between adjacent SNPs across the chicken autosomes studied here ranged from 0.34 to 0.40 in the pure lines but was only 0.24 in the crossbred populations, with 28.4% of adjacent SNP pairs having an *r*^2^ higher than 0.3. Compared with the pure lines, the crossbred populations consistently showed a lower level of LD, smaller haploblock sizes and lower haplotype homozygosity on macro-, intermediate and micro-chromosomes. Furthermore, correlations of LD between markers at short distances (0 to 10 kb) were high between crossbred and pure lines (0.83 to 0.94).

**Conclusions:**

Our results suggest that using crossbred populations instead of pure lines can be advantageous for high-resolution QTL (quantitative trait loci) mapping in GWA studies and to achieve good persistence of accuracy of genomic breeding values over generations in genomic selection. These results also provide useful information for the design and implementation of GWA studies and genomic selection using crossbred populations.

**Electronic supplementary material:**

The online version of this article (doi:10.1186/s12711-015-0098-4) contains supplementary material, which is available to authorized users.

## Background

Genome-wide association (GWA) studies and genomic selection are currently widely applied in animal genetics research and animal breeding programs. Using thousands of genetic markers, the ultimate goal of most GWA studies is to find causal polymorphisms that affect a phenotype [1], whereas a reliable prediction of the total genetic value of selection candidates is the major goal of genomic selection [2]. Despite their different aims, the success of these two approaches depends primarily on the level of linkage disequilibrium (LD) between markers and causal polymorphisms [3,4]. For example, long-distance LD is useful in GWA studies when using a relatively low-density set of markers [5-7]. However, mapping resolution is expected to be lower when LD extends over long distances because multiple markers across a wide chromosomal region may be in LD with the causal polymorphism and all show significant associations with the trait. Conversely, for the opposite reason, a low level and extent of LD can be useful for high-resolution association mapping.

Progress in next-generation sequencing and high-density single nucleotide polymorphism (SNP) genotyping technologies offer unprecedented opportunities for detecting causal polymorphisms or achieving high accuracies of prediction in genomic selection [8-12]. However, taking full advantage of these new technologies may be limited in livestock populations due to the high level and extent of LD. Although LD extends over long distances in most livestock populations, comparisons of LD patterns between populations show that shared haplotype segments are much shorter when the population consists of multiple purebred populations [13-15]; this indicates that LD decays more quickly in multi-breed or crossbred populations than in purebred populations. Therefore, in cases where LD does not extend over long distances, multi-breed and crossbred populations can be potentially useful for fine mapping and identification of causal polymorphisms. Furthermore, as explained in the Discussion section, using a crossbred population as reference population in genomic selection can also be advantageous, particularly in livestock species with crossbreeding programs, such as poultry, pigs and beef cattle.

In this study, our aim was to characterize the consistency of LD and differences in LD between crossbred and their component purebred populations. As for previous studies on LD in layer [16] and village chickens [17], we used the Illumina 60K chicken SNP panel [18] which contains over 10 times more SNPs than that of most other studies on LD in chickens [19-23].

## Methods

### Data

**Figure 1:**
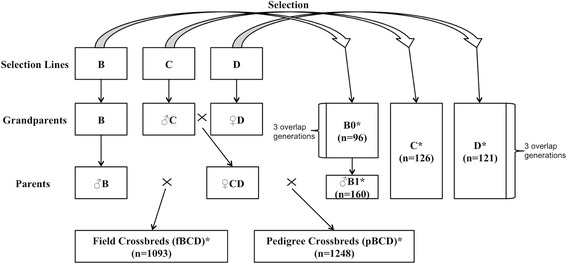
**Schematic representation of the broiler population structure.** Genotyped pure lines and crossbred individuals were sampled from pedigree pure lines (right) and two broiler-crossbreeding programs (bottom). Among the pedigree pure lines, line B is a male line and both lines C and D are female lines. All genotyped birds (indicated by “*”) were sampled from male flocks. The genotyped birds from male and female lines were elite sires randomly sampled from three overlapping generations. A random sample of the B0 elite sires’ progeny (B1) was also genotyped. The field crossbred chickens (fBCD) were end-product meat birds, whereas the pedigree crossbred chickens (pBCD) were produced for the genetic evaluation of B1 sires. The CD mothers of the field and pedigree crossbred chickens were different but from the same generation of CD parents. The line B fathers of field and pedigree crossbred chickens were diverged for two to three generations.

A total of 2844 individuals were genotyped using the Illumina 60K chicken SNP array [18]. All genotyped birds were sampled from male flocks and included 2341 crossbred and 503 purebred chickens. Among the 503 genotyped purebred chickens, 256 were sampled from a male line, i.e. line B, and 126 and 121 chickens were sampled from two female lines, i.e. lines C and D, respectively (there was no line A in this study). Individuals that were genotyped from the female lines were elite sires that were randomly sampled from three overlapping generations. Only a portion of the chickens that were genotyped from the male line (B0; n = 96) were elite sires that were also sampled from three overlapping generations; another set of genotyped chickens (B1; n = 160) was a random sample of the progeny of the B0 elite sires (Figure 1).

All 2341 genotyped crossbred chickens were produced by a three-way cross of B × [C × D], in which males of line B were mated with CD crossbred females, which is a crossbred female product produced by a two-way C × D cross, in which males of line C were mated with females of line D. Crossbred individuals were sampled from two broiler chicken populations: (i) broiler chickens from the field (end product meat birds), which will be referred to as field crossbred chickens (or fBCD, n = 1093) and (ii) broiler chickens from a pedigree house (produced for genetic evaluation of the pedigree B1 sires), which will be referred to as pedigree crossbred chickens (or pBCD, n = 1248) (Figure 1).

To assess the extent to which the LD pattern in crossbred populations can be predicted based on the genotypes of their component pure lines, we created a combined population by simply combining the genotype data of a random sample of 200 chickens of line B, 100 of line C and 100 of line D. The 2:1:1 proportion of lines B:C:D was used to mimic the expected genetic contribution of these lines to the autosomes of crossbred individuals. This combined population will be referred to as the combined BCD (or cBCD) population.

The Illumina 60K SNP chip contains 57636 SNPs [18]. In this study, we used only SNPs with assigned positions on autosomes (based on the latest reference genome, *Gallus gallus* 4.0 UCSC, May 2012). Within each pure line and crossbred population, we discarded SNPs with a call rate less than 90%, Mendelian inconsistency greater than 0.001 and minor allele frequency (MAF) less than 0.05. Also, SNPs that strongly deviated from Hardy–Weinberg equilibrium (*p* value < 0.001) in the pure lines were discarded, as well as SNPs on chromosome 16 and two linkage groups because there were too few SNPs in the 60K SNP panel for these chromosomes.

### Haplotype and haploblock analyses

Determining haplotype phase and frequency is necessary to estimate LD and can provide useful information about breed-specific haplotypes and the history of artificial selection. We used BEAGLE (Version 3.3.2) [24] to infer haplotype phase for each genotyped individual in each population. As in Badke et al. [8], we set BEAGLE to run 100 iterations of the phasing algorithm and to sample 100 haplotype pairs for each individual per iteration.

In theory, haplotype homozygosity is defined as the likelihood of randomly sampling two identical haplotypes from a population, which is calculated as the sum of squares of haplotype frequencies [25]. Based on the results of the haplotype phases obtained with BEAGLE, haplotype homozygosity was estimated using haplotype frequencies for 250-kb sliding windows, with a step size of 25 kb. For each population, Haploview (Version 4.2) [26] was used to define haplotype blocks (haploblocks) with the built-in algorithm suggested by Gabriel et al. [27]. In this model, the confidence interval of observed values of LD measured by D' was estimated to determine the upper and lower confidence bounds of D’ (5% tails of the overall probability distribution of D'), and the blocking structure was determined by defining SNP pairs to be in “strong LD” if the upper confidence bound was above 0.98 and the lower bound was above 0.7 [27].

### Estimation of linkage disequilibrium

We calculated *r* using the equation below and used its square, *r*^2^, to measure LD between marker pairs that are separated by less than 5 Mb on each chromosome:

$$ {r}_{ij}=\frac{f(MN)-f(M)f(N)}{\sqrt{f(M)f(m)f(N)f(n)}} $$

where *r*_*ij*_ is the correlation between alleles at SNP *i* (alleles *M* and *m*) and alleles at SNP *j* (alleles *N* and *n*); *f(MN)* is the observed frequency of haplotype *MN*, which can be simply obtained from the phasing results; and *f(M), f(m), f(N)* and *f(n)* are observed frequencies of alleles *M, m, N* and *n*, respectively [28].

Previous studies on LD in chickens showed that the extent of LD over physical distances varies greatly among the different categories of avian chromosomes: macro-chromosomes (GGA1 to 5, GGA for Gallus gallus chromosome), intermediate chromosomes (GGA6 to 10) and micro-chromosomes (GGA11 to 38) [29]. Thus, we estimated LD separately for each category of chromosomes within each population. To visualize the LD pattern for each category of chromosomes in different populations, *r*^2^ values were ordered in ascending order based on the physical distance between the corresponding SNP pairs, and then a rolling average of LD was calculated as the arithmetic mean of all *r*^2^ values for SNP pairs in 25-kb intervals and plotted against physical distance between SNPs.

### Estimation of consistency of LD

Consistency of LD between two populations was calculated as the correlation of *r* between SNP pairs. We used the SNPs that were common to the populations to estimate the consistency of LD as the correlation *r*_*ij*_ between the same pairs of SNPs within a given interval in two populations. For simplicity, this will be referred to as the correlation of *r*. To visualize and compare the correlation of *r* among different pairs of populations, the pairwise correlation of *r* was estimated separately for each category of chromosomes in 50-kb non-overlapping intervals and plotted against physical distance.

## Results

### Marker statistics

The numbers of SNPs that remained after quality control and were used in subsequent analyses for pure lines B, C and D, and field crossbred (fBCD) and pedigree crossbred populations (pBCD) ranged from 36379 to 43653 and are in Table 1. There were 26350 common SNPs in these five populations. SNPs that were evaluated in the combined BCD (cBCD) population were the same as those included in the field crossbred population.

**Table 1:** **Quality control criteria and number of SNPs discarded in each population**

**Quality control**	**Population** ^**1**^					
	**All**	**B**	**C**	**D**	**fBCD**	**pBCD**
Chromosomes not included	4522					
Mendelian inconsistency	1456					
SNPs not called^2^		706	978	978	823	839
Monomorphic SNPs		7907	9888	7768	3467	3582
SNPs with a call rate < 0.9		550	154	230	510	448
SNPs with a MAF < 0.05		4914	4096	4300	3205	3149
HWE (*p* value < 0.001)		121	163	194	NT^3^	NT^3^
SNPs in use		37460	36379	38188	43653	43640
Common SNPs	26350					

^**1**^B: line B; C: line C; D: line D; fBCD: field crossbred chickens; pBCD: pedigree crossbred chickens; ^2^SNPs that were genotyped but not called; ^3^NT: the Hardy-Weinberg equilibrium test was not applied to crossbred chickens.

**Figure 2:**
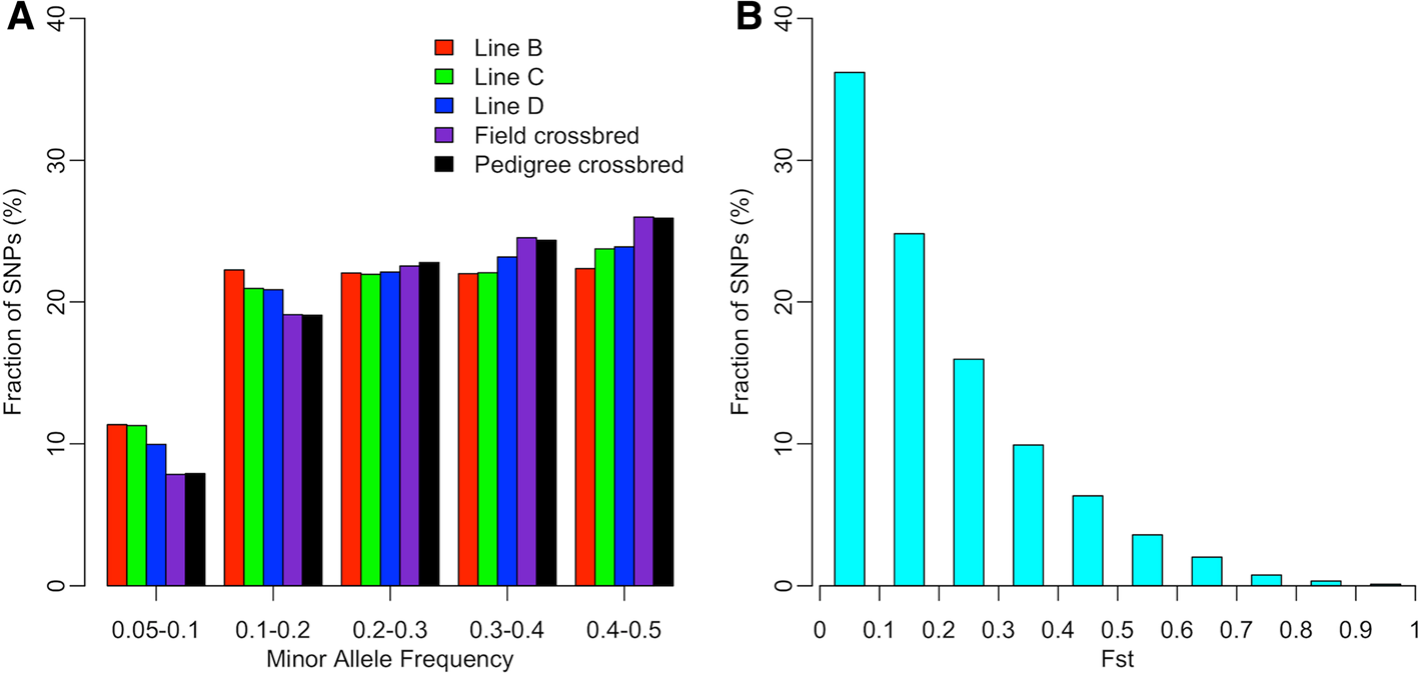
**Distribution of minor allele frequency and F**_**st**_ **of SNPs. A**: Distribution of MAF of SNPs after quality control in each population. Each population is represented by a different color. **B**: Distribution of F_st_ of common SNPs to the three purebred populations.

Distributions of MAF for SNPs after quality control are in Figure 2A for each population. More than 65% of SNPs in the three purebred populations and more than 70% of SNPs in the crossbred populations had a MAF greater than 0.2. MAF distributions were mostly uniform for MAF greater than 0.05. As expected, the number of SNPs with a high MAF was larger for the crossbred populations than for each of the pure lines. In addition, F_st_ [30] were estimated among the three purebred populations for all common SNPs after quality control, and their distributions are in Figure 2B. The average F_st_ was greater than 0.20, which suggests that there was substantial genetic differentiation among these purebred populations [31].

### Linkage disequilibrium

**Figure 3:**
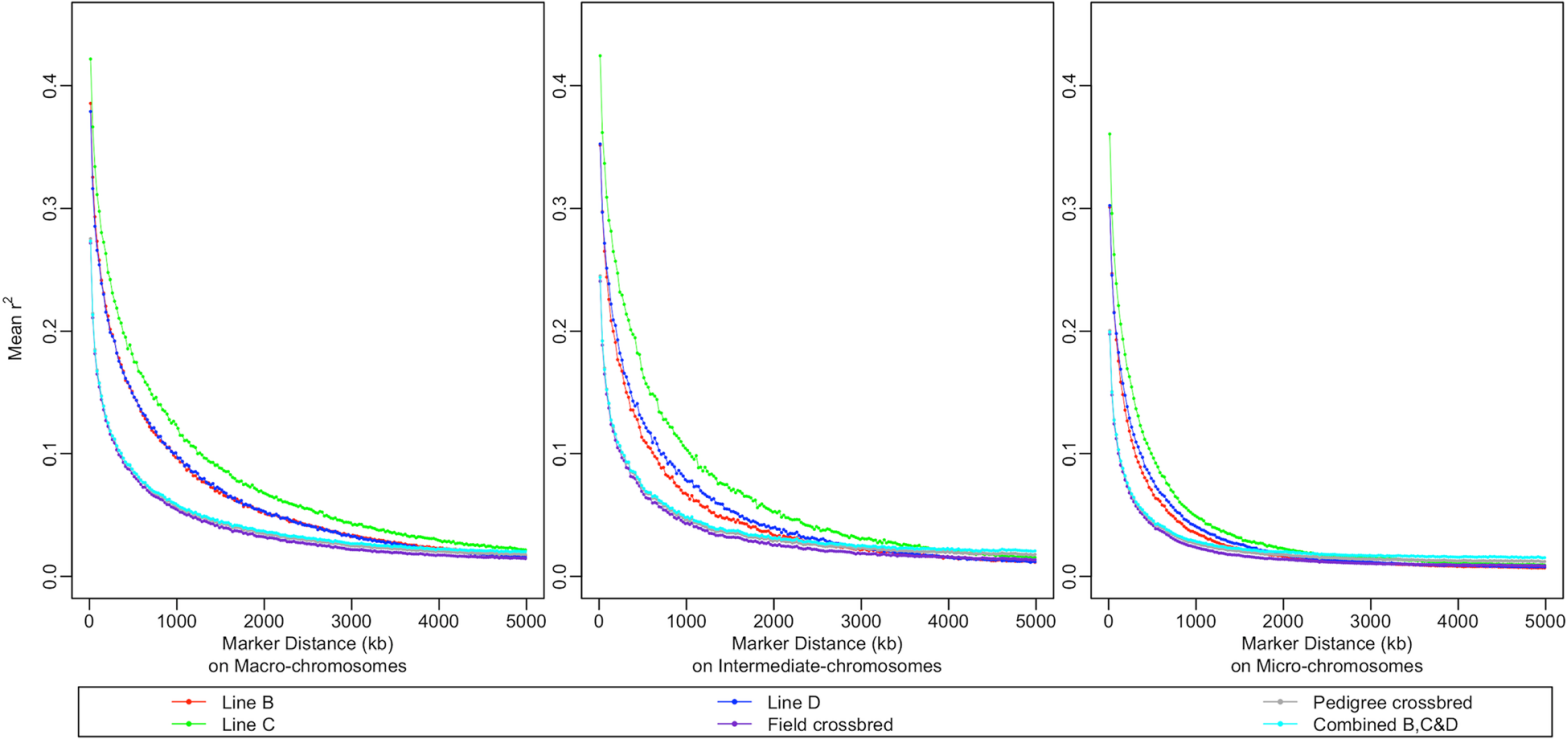
**Decay of linkage disequilibrium with distance on different categories of chromosomes in different populations.** Each population is represented by a different color. Each point in the plots represents the mean *r* ^*2*^ of marker pairs in a 25-kb interval. Points representing field and pedigree crossbred and combined BCD populations (purple, grey and cyan, respectively) are almost overlapping and difficult to distinguish in most areas.

As shown in Figure 3, LD declined as the distance between markers in all populations increased, and *r*^*2*^ converged to 0.02 approximately at around 2, 4 and 5 Mb on micro-, intermediate, and macro-chromosomes, respectively. At marker interval distances smaller than 1 Mb, LD differed considerably between crossbred and purebred populations and also between chromosome categories. Micro-, intermediate, and macro-chromosomes showed the lowest, second lowest and highest mean *r*^2^, respectively, across all populations (Figure 3). The crossbred populations and line C displayed the lowest and the highest mean *r*^2^, respectively. The mean *r*^2^ of lines B and D were similar but lower than that of line C. Compared with the pure lines, the distance at which *r*^2^ decayed below 0.2 (D_0.2_) was considerably smaller in the crossbred populations; in the crossbred populations, D_0.2_ was equal to ~50, ~25, and ~15 kb for the macro-, intermediate, and micro-chromosomes, respectively while in the pure lines, D_0.2_ was greater and equal to 225, 150, and 80 kb. Line C showed the largest D_0.2_ for all three categories of chromosomes.

The mean *r*^2^ between adjacent SNPs across all autosomes studied here ranged from 0.34 to 0.40 in the pure lines but was on average only equal to 0.24 in the crossbred populations. Due to the different densities of SNPs on each chromosome in the 60K SNP chip, mean *r*^2^ values were similar in the three categories of chromosomes. Furthermore, in the pure lines, at least 53.2% of adjacent SNP pairs had an *r*^2^ greater than 0.2 and 42.2% had an *r*^2^ greater than 0.3, but in the crossbred populations, only 39.5% and 28.4% showed an *r*^2^ greater than 0.2 and 0.3, respectively.

**Figure 4:**
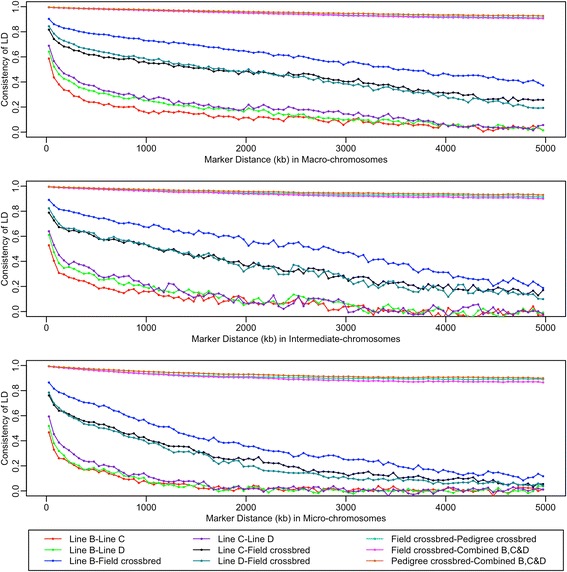
**Correlation of LD on different chromosome categories among the pure lines and crossbred populations.** Each population is represented by a different color. Each point in the plots represents the mean correlation of *r* in a 50-kb interval. Points representing the correlations of *r* between field and pedigree crossbred populations and combined BCD populations are almost overlapping and difficult to distinguish in some areas.

The two crossbred populations and the combined BCD population showed almost the same level of LD (Figure 3) and very high correlations of *r* (Figure 4) at all distances between SNPs studied here. Thus, only results of the field crossbred population *vs.* pure lines are presented here.

### Haploblock and haplotype homozygosity

**Figure 5:**
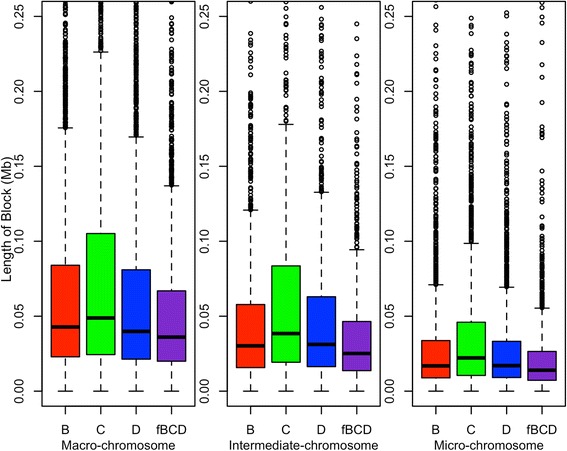
**Length of haploblocks in different categories of chromosome in different populations.** B: line B; C: line C; D: line D; fBCD: field crossbred population. Different populations are represented by different colors. The ends of the whiskers represent the lowest datum within 1.5 IQR (interquartile range) of the lower quartile, and the highest datum within 1.5 IQR of the upper quartile.

The statistics of haploblock distributions in the different populations are in Table 2 and Figure 5. After quality control, more than 62% of SNPs formed haploblocks in the pure lines but only 30.6% in the field crossbred population. Also, the field crossbred population had the lowest genome coverage in haploblocks and the smallest overall median haploblock size. Moreover, nearly half (49.1%) of the haploblocks in the field crossbred population were slightly smaller than 25 kb, compared with 42.6%, 36.6% and 43.5% in the pure lines B, C and D, respectively. Line C had the largest genome coverage in haploblocks and showed the largest median haploblock size. Among the three chromosome categories and in each population, macro-chromosomes had the largest average length of haploblocks, followed by the intermediate and micro-chromosomes [See Additional file 1: Table S1]. All these results were consistent with the LD patterns observed in these populations.

**Table 2:** **Summary statistics of haploblock structure across different populations**

**Statistics**	**Population** ^**1**^			
	**B**	**C**	**D**	**fBCD**
Median block size (kb)	30.8	37.2	29.6	25.7
Maximum block size (kb)	3521.9	3527.6	4226.0	2737.2
Genome coverage (Mb)^2^	446.7	485.8	401.4	229.0
TSNPs^3^	26293	25720	23763	13375
BSNPs (%)^4^	70.2	70.7	62.2	30.6
Mean ± SD nBSNPs^5^	3.5 ± 3.4	4.2 ± 4.6	3.5 ± 3.9	2.6 ± 2.1
Max nBSNPs^6^	86	89	118	86

^1^B: line B; C: line C; D: Line D; fBCD: field crossbred chickens; ^2^genome coverage with all haploblocks; ^3^total number of SNPs that form haploblocks; ^4^percentage of SNPs that form haploblocks; ^5^mean and standard deviation of number of SNPs that form haploblocks; ^6^maximum number of SNPs that form haploblocks.

**Figure 6:**
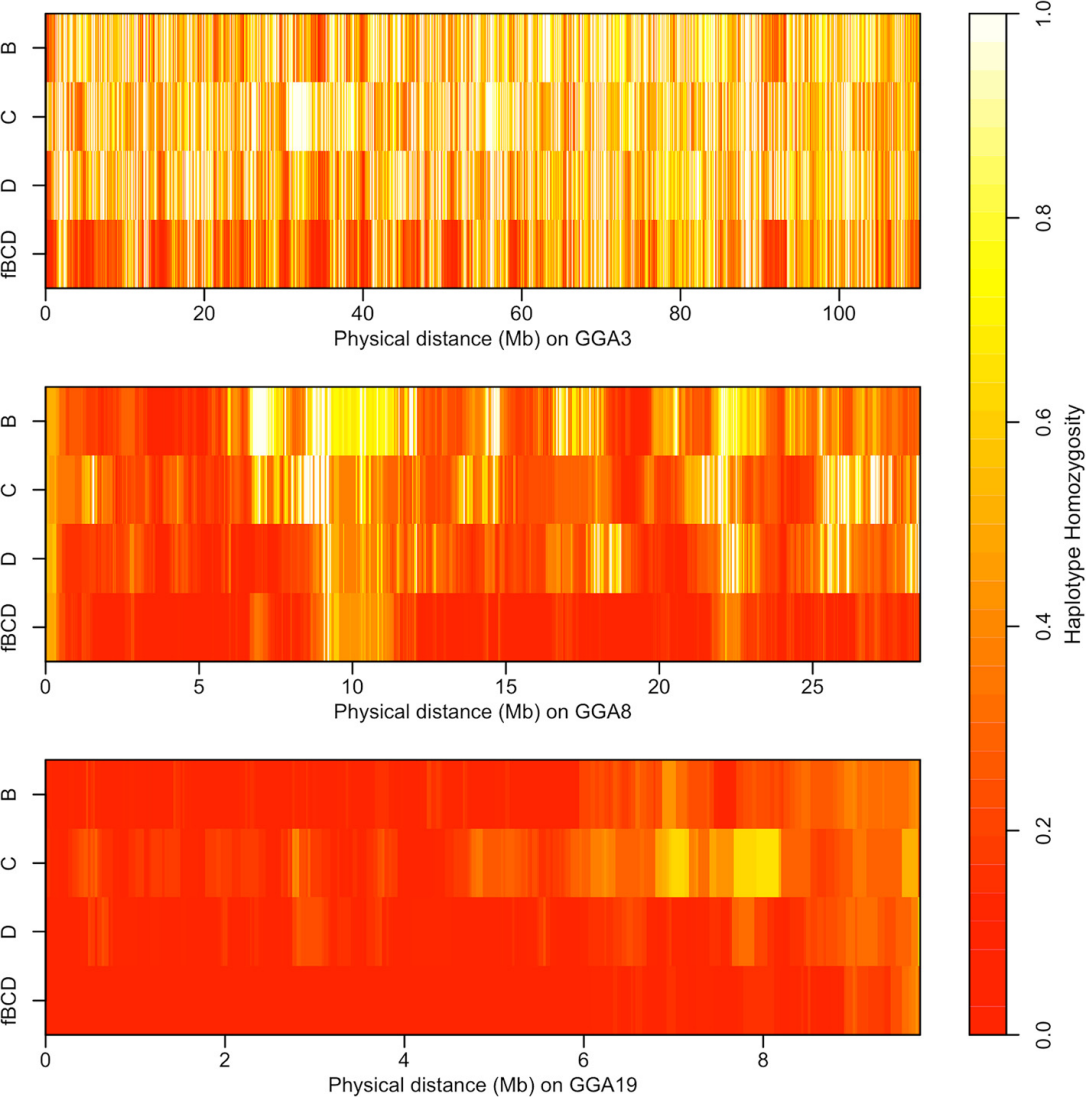
**Haplotype homozygosity on chicken chromosomes GGA3, GGA8 and GG19.** B: line B; C: line C; D: line D; fBCD: field crossbred population. The end position on each chromosome represents the physical position of its last SNP on the Illumina 60K chicken SNP panel. Each bin in the plots has a size of 25 kb and represents haplotype homozygosity of a 250-kb sliding window with a step size of 25 kb on each chromosome.

Haplotype homozygosity (HH) was measured over sliding windows of 250 kb. Results for chromosomes 3, 8 and 19 are in Figure 6 and represent macro-, intermediate, and micro-chromosomes, respectively. Among all the populations, crossbred populations showed a lower average HH than purebred populations for all chromosome categories, with most HH values being less than 0.1. Differences between populations were not very obvious for the micro-chromosomes, because the extent of HH with a window size of 250 kb was very small for micro-chromosomes compared to macro-chromosomes. Although the overall HH pattern was consistent with the results of LD analyses in these populations, the local HH pattern on each chromosome varied among the four populations.

### Consistency of LD

The correlation of *r* measures the degree of agreement of the direction and level of LD for SNP pairs between two populations [8,9]. In principle, these correlations can range from −1 to 1: a high positive value indicates high LD and the same haplotype phase in the two populations, and a high negative value results from a high LD between two markers but with the opposite phase in the two populations [12].

It should be noted that in this study correlations of *r* were high and positive (>0.99) between field and pedigree crossbred populations and also between either of these real crossbred populations and the combined BCD population at distances between markers less than 50 kb (see Figure 4). The correlation gradually decayed as the distance between markers increased but still remained high (0.87 to 0.93), even if SNPs were about 5 Mb apart.

Our results show that correlations of *r* between the pure line C and field crossbred population (0.86 to 0.92; < 10 kb) and between the pure line D and field crossbred population (0.83 to 0.88; < 10 kb) were generally similar but lower than those between the pure line B and field crossbred population (0.91 to 0.94; < 10 kb). Among the pure lines, the correlation of r between the two female lines was slightly higher than that between the male line and either of the female lines.

As with the decay of LD, correlations of *r* decreased with physical distance and this decrease was greater for the smaller chromosomes. For example, correlations of *r* between the pure line B and field crossbred population were 0.94, 0.94 and 0.91 in the interval of 0 to 10 kb on macro-, intermediate and micro-chromosomes, respectively, but decreased to 0.39, 0.21 and 0.13, respectively, at an interval of approximately 5 Mb.

## Discussion

Studies of LD in farm animals have been mostly limited to purebred populations and there is limited information about the extent of LD in crossbred populations and the consistency of LD phase between crossbred populations and their parental pure lines [10]. In this study, we characterized the consistency of phase and level of LD between crossbred broiler chickens and their parental pure lines. The crossbred chickens in our study were a three-way cross of B × [C × D] that was produced using three pure lines B, C and D, which contributed 50, 25, and 25% of genetic material, respectively, to the autosomes of the crossbred animals. Our analyses used SNP genotypes on 27 chicken autosomes (GGA1 to 28, excluding GGA16 and other micro-chromosomes because the 60K SNP chip did not include enough markers on these chromosomes). To our knowledge, there is no published report that characterizes LD in crossbred chicken populations and that compares the consistency of phase and level of LD between crossbred and their parental pure lines.

### Rapid decay of LD in crossbred populations and micro-chromosomes

The level and pattern of LD differed between the populations used in our study. The two field and pedigree crossbred populations had very similar levels of LD for all physical distances. However, compared with the pure lines, crossbred populations showed a small extent and rapid decay of LD by distance for all three chromosome categories. For example, for the macro-chromosomes, the mean *r*^2^ for SNPs that were 0 to 10 kb apart was 0.32 in the field crossbred population but greater than 0.44 in the pure lines, and the mean *r*^2^ dropped to less than 0.2 at distances between SNPs of ~50 kb in the field crossbred population, whereas in the pure lines, this drop was observed for much greater distances (~225 kb). Similarly, the extent of LD was smaller in crossbred beef cattle than in purebred Angus and Charolais cattle [10]. The rapid decay of LD by distance in crossbred populations can be useful for high-resolution mapping of causal polymorphisms. Indeed, if the extent of LD is small, it is less likely that SNPs far away from a causal polymorphism will be in LD with the polymorphism, which confines associations to SNPs that are in close vicinity of the causal polymorphism, thereby increasing map resolution. Moreover, when using a higher SNP density, the small extent of LD in crossbred populations may be an advantage for genomic selection because the tight linkage between causal polymorphisms and adjacent SNPs is less likely to be broken down by recombination, and therefore the accuracy of genomic predictions will persist over more generations [32].

An average LD (*r*^2^) greater than 0.2 [3,33,34] or 0.3 [11,35] between adjacent SNPs has been recommended to detect SNPs associated with causal polymorphisms or to achieve a reasonable accuracy of prediction in genomic selection. Although in the pure lines at least 53.2% of adjacent SNP pairs of the 60K SNP panel had an *r*^2^ greater than 0.2 and 42.2% had an *r*^2^ greater than 0.3, in the field crossbred population, only 28.4% of adjacent SNP pairs showed an *r*^2^ greater than 0.3, which suggests that a higher density SNP panel would be an advantage for GWA studies or to implement genomic selection in commercial crossbred populations.

In our study, the extent of LD varied greatly between chromosome categories in different populations and decreased as the distance between SNPs increased (Figure 3). Consistent with previous studies [16,20,23], our results showed that small-size autosomes had less LD than large-size autosomes. The differences in LD between small- and large-size autosomes have been attributed mainly to differences in recombination rates per unit of physical distance, with micro-chromosomes showing the largest recombination rate per Mb (6.4 cM/Mb), followed by the intermediate chromosomes (3.9 cM/Mb), and then macro-chromosomes (2.8 cM/Mb) [29].

**Figure 7:**
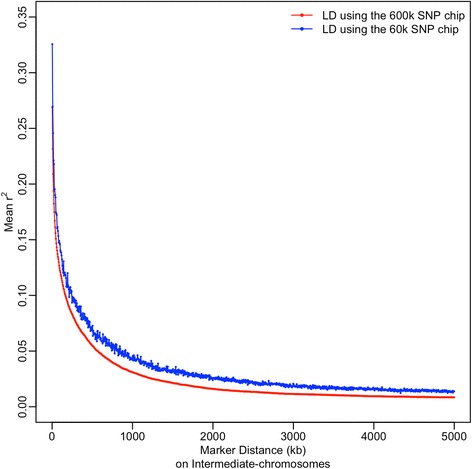
**Decay of linkage disequilibrium with distance on intermediate chromosomes in field crossbred chickens using different SNP arrays.** The results of LD using the 600K and 60K SNP arrays are represented by red and blue color, respectively. Each point in the plot represents the mean *r* ^*2*^ of marker pairs in a 5-kb interval.

It was noted that SNP ascertainment bias (Figure 2A) could be an important factor affecting our results of LD analysis based on SNPs in the Illumina 60K chicken SNP array. SNP ascertainment bias of genotyping arrays is mainly related to the protocol used to identify SNPs and to the sampling of a limited number of non-random individuals for their detection. In general, this leads to overestimation of LD [36,37]. For example, for the Illumina 60K chicken SNP array, only four commercial breeding lines (two broilers and two layer lines) were used for SNP detection, and SNPs were identified by sequencing DNA pooled from 25 individuals from each of these commercial breeding lines [18]. Furthermore, only SNPs with medium to high MAF were selected. Each of these limitations can be a potential factor contributing to SNP ascertainment bias in our data obtained using the Illumina 60K chicken SNP array. Although SNP ascertainment bias cannot be avoided when using genotyping arrays, for the purpose of comparison, we estimated LD on intermediate chromosomes in 72 crossbred chickens that were genotyped using the recently available Affymetrix 600K chicken SNP array. This array was designed by sequencing more individuals, i.e. 243 chickens from 24 chicken lines, including 15 commercial lines (broilers or layers), inbred layers and one unselected layer line [38]. Therefore, compared to the 60K SNP array, results from the 600K SNP array should be less affected by SNP ascertainment bias. As expected, our results showed that the LD (*r*^2^) measured at distances up to 5 Mb was slightly lower with the 600K SNP array than with the 60K SNP array. The average difference in LD at distances up to 1 Mb was, however, less than 0.016 (i.e. 19.1% reduction in average LD when using the 600K SNP array), and the differences became smaller and more stable at larger distances (Figure 7). Thus, although SNP ascertainment bias cannot be avoided when using genotyping arrays, we believe our results and conclusions on differences in LD patterns in crossbred and pure line chickens are reliable.

### Small haploblock size in crossbred populations

Consistent with the results of the LD analysis, results of the haploblock analyses showed that the crossbred populations had the lowest genomic coverage (229.0 Mb) in haploblocks and the smallest average number of SNPs forming haploblocks. Given the size of the autosomes (~918.9 Mb; GGA1 to 28 without GGA16; *Gallus gallus* 4.0, November 2011), this means that haploblocks covered only 24.9% of the genome of the crossbred populations, which is nearly half that of the pure lines. In a study using commercial chickens, the genome coverage in haploblocks ranged from 337.1 to 599.4 Mb, with an F1 cross between two layer populations showing a lower genome coverage (337.1 Mb) than the layer pure line [16]. Although the pure lines in this study were not the parental lines of this F1 cross, the low genome coverage in haploblocks in this two-way layer cross is consistent with what we observed in the three-way broiler cross.

The percentage of SNPs forming haploblocks differed between populations (Table 2). Only 31.4% of SNPs formed haploblocks in the field crossbred population, compared to more than 60% in the three pure lines. This finding shows that, because of the small extent of LD, most markers did not form haploblocks in the crossbred populations. The small percentage of SNPs forming haploblocks and the small haploblock size in the crossbred populations also indicate that the 60K SNP chip used in our study does not have an adequate SNP density for high-resolution characterization of the haplotype structure in crossbred chickens. Megens et al. [23] investigated the LD and haplotype diversity on four ~1-cM regions on macro- and micro-chromosomes and suggested that whole-genome marker assays would need to contain at least 100 K informative SNPs to exploit haplotype information in commercial chicken populations. Consistent with this, a study on the haplotype structure of traditional and village chickens suggested using at least 90 to 110 K SNPs to construct a whole-genome haplotype map for these populations [17]. Therefore, the recently available 600K SNP chicken panel [38] is likely to provide a higher resolution haplotype map in crossbred chickens.

Haplotype homozygosity is a measure of haplotype diversity in a population. In a previous study, a relatively small number (1 to 7) of haplotypes accounted for most of the haplotype diversity (>90%) found on the macro-chromosomes, but not on the micro-chromosomes [23]. These results are consistent with our findings across all populations. Within 250-kb windows, the pure-line chickens showed limited haplotype sharing, which is consistent with a moderate correlation of *r* (<0.5) at the same distance between these populations (Figure 4). In some chromosomal regions, high levels of HH extended over longer distances in one pure line but not in the others. Because one of the key characteristics of positive selection is the presence of unusually long-range HH [39], these differences in HH patterns could be considered as evidence of recent positive selections in these pure lines. Therefore, it would be interesting to conduct an analysis of selection sweeps in these pure lines.

### Consistency of LD from pure lines to crossbreds

Because of the pyramidal structure of conventional chicken breeding programs, there is approximately four years of lag time from pedigree pure line birds to end-product crossbred birds [40]. To assess if LD persists between pedigree pure lines and commercial crossbred birds, we estimated the correlation *r* between pure lines in the pedigree program (top of the pyramid) and crossbred chickens that were sampled from the field (bottom of the pyramid). Correlations of *r* were high (0.83 to 0.94) between these populations for closely spaced SNPs (0 to 10 kb) but these correlations decreased as the distance between SNPs increased; correlations dropped by 4 to 9% from 0 to 10 kb to 10 to 50 kb distances between markers across the three chromosome categories. In our study, the 60K SNP panel provided an average marker spacing of one informative SNP per ~25 kb; therefore, it is expected that using a higher density SNP panel (such as the recently available 600K SNP array) will increase the accuracy of genomic selection of pure lines for crossbred performance. This conclusion is consistent with results from two simulation studies [32,41] that showed that training in crossbred populations led to slightly less accurate predictions of true breeding values of selection candidates in purebred populations compared with training only in the selected purebred population. However, by increasing the density of the SNP panel, differences in accuracies became much smaller.

The correlation of *r* between crossbred populations and pure lines differed in the three chromosome categories, with macro-chromosomes showing the highest levels of correlation and micro-chromosomes showing the lowest. These results indicated that, in GWA studies or in genomic selection programs, micro- and intermediate chromosomes would require a higher SNP density per kb than macro-chromosomes. In our study, each pure line showed a different level of correlation of *r* with the crossbred populations and, of all comparisons, this value was the highest between line B and the crossbred populations. As mentioned before, line B was the terminal male line for crossbred chickens B × [C × D], which means that this line contributed 50% of genetic material to the autosomes of crossbred individuals; whereas the female lines C and D were expected to contribute each 25%. As for female lines, the correlation of *r* was greater between line D and the crossbred populations than between line C and the crossbred populations, which could be due to a greater correlation of *r* between lines D and B than between lines C and B.

### Consistency of LD phase among pure lines

The correlation of *r* can reflect the relative degree of similarities and divergences between purebred animals. In a large-scale genome-wide survey of SNP variation in cattle breeds, the correlation of *r* declined as the divergence between breeds increased [42]. In our study, the correlation of *r* between female lines was slightly but consistently higher than that between female and male lines, which suggests that the two female lines may share a more similar genetic background than each of these lines does with the male line. This is consistent with the fact that male and female lines originated from different breeds, i.e. the male line from Cornish, a meat type breed, and the female lines from dual-purpose breeds. Furthermore, since selection goals of male and female lines are different, this may have contributed to the similarity between female lines and to the divergence between the female lines and the male line. Unlike the male line, which was selected primarily for growth-related traits, the female lines were selected for both reproductive and growth traits.

Using markers on chromosomes 1 and 4, Andreescu et al. [21] estimated correlations of LD within 500 kb among nine purebred chicken lines from a commercial broiler breeding company; the correlations over all pairs of lines ranged from 0.21 to 0.94, with an average correlation of 0.52. Badke et al. [8] reported a correlation of LD for distances between markers less than 10 kb that was equal to 0.92 between Landrace and Yorkshire breeds and 0.87 between these breeds and the Duroc breed; these values decreased to 0.41 to 0.57 for distances between markers around 1 Mb. Moreover, a study in cattle found that the correlation of LD for distances between markers less than 10 kb was 0.97 between Dutch black-and-white Holstein-Friesian *vs.* Dutch red-and-white Holstein-Friesian and New Zealand Friesian *vs.* Zealand Jersey [9]. In our study, none of the chromosome categories reached this high level of consistency of LD between pure lines for distances between markers less than 10 kb, and correlations of *r* ranged from 0.73 to 0.82 for distances between markers less than 10 kb, even on macro-chromosomes. It is likely that the genetic diversity of the chicken lines in our study was greater (average F_st_ > 0.20) than that of both the cattle and pig breeds used in the aforementioned studies. Another possible explanation for this difference is the overall higher recombination rates per unit of physical distance on chicken chromosomes compared with the average ratio of 1 cM/1 Mb in mammalian livestock animals.

### Consistency of LD between crossbred populations and the combined BCD population

To assess the extent to which the LD pattern in crossbred populations can be predicted using the genotypic information of their component pure lines, we created a combined BCD population (see Methods section) and studied the differences in LD between this hypothetical population and the actual crossbred populations. Across all three chromosome categories, levels of LD were almost the same in these crossbred and the combined BCD populations (Figure 3). This was also reflected by the consistency of LD between these populations, since the correlations of *r* were very high (>0.99) for distances between markers less than 50 kb between the crossbred and the combined BCD populations, and decayed gradually as the distance between markers increased, but still remained high (0.87 to 0.93) for markers that were about 5 Mb apart (see Figure 4). These results indicate that by using only genotype information on the pure lines, one can predict the LD in crossbred populations with very high accuracy, as well as the correlation *r* between crossbred populations and their component pure lines.

## Conclusions

In conclusion, our study characterized the extent and consistency of LD in commercial broiler populations from different angles and showed that, between crossbred populations and their component pure lines, the consistency of the level and phase of LD for short distances between markers (0 to 10 kb) is remarkably high. Compared with the pure lines, the crossbred populations showed a considerably lower level of LD and a smaller haploblock size, which suggests that using crossbred animals as a reference population can be an advantage for high-resolution mapping of causal polymorphisms in GWA studies and to achieve better persistence of the accuracy of genomic estimated breeding values over generations in genomic selection programs. However, our results also suggest that a higher SNP density, particularly on micro-chromosomes, is necessary to take full advantage of crossbred populations in GWA studies or in genomic selection programs. Finally, our results prove that LD for short and long distances between markers and haplotype phase for short distances between markers in a crossbred population can be predicted with very high accuracy using genotype information of its parental pure lines.

## Additional file

Additional file 1: Table S1. Summary statistics of haploblock structure for different chromosome categories. Statistics of haplotype structure are presented for different chromosome categories in chicken genome. ^1^B: line B; C: line C; D: Line D; fBCD: field crossbred chickens; ^2^median haploblock size; ^3^maximum haplolock size; ^4^total number of SNPs that form haploblocks.
