# Supplementary material for: Linkage disequilibrium in crossbred and pure line chickens
Source: Genet Sel Evol. 2015 Feb 26;47(1):11. doi: 10.1186/s12711-015-0098-4 (PMC4341223; doi:10.1186/s12711-015-0098-4)
Supplement: Additional file 1: Table S1. — Summary statistics of haploblock structure for different chromosome categories. Statistics of haplotype structure are presented for different chromosome categories in chicken genome. 1B: line B; C: line C; D: Line D; fBCD: field crossbred chickens; 2median haploblock size; 3maximum haplolock size; 4total number of SNPs that form haploblocks. [file 12711_2015_98_MOESM1_ESM.docx]

**Table S1** Summary statistics of haploblock structure on different chromosome types

| Chromosome Type | Statistics | Populations^1^ | | | |
| --- | --- | --- | --- | --- | --- |
|  |  | B | C | D | fBCD |
| Macro | MedBS^2^ (kb) | 42.8 | 48.8 | 40.0 | 36.1 |
|  | MaxBS^3^ (kb) | 3,521.9 | 3,527.6 | 4,226.0 | 2,737.2 |
|  | TSNPs^4^ | 13,535 | 13,353 | 12,324 | 6,956 |
| Intermediate | MedBS^2^ (kb) | 30.2 | 38.4 | 31.2 | 25.1 |
|  | MaxBS^3^ (kb) | 1,810.0 | 1,868.0 | 1,640.0 | 1,421.0 |
|  | TSNPs^4^ | 4,402 | 4,255 | 4,007 | 2,108 |
| Micro | MedBS^2^ (kb) | 16.9 | 22.2 | 17.0 | 14.0 |
|  | MaxBS^3^ (kb) | 1,330.0 | 975.0 | 774.2 | 794.3 |
|  | TSNPs^4^ | 8,356 | 8,112 | 7,432 | 4,311 |
